# AI enabled, mobile soil pH classification with colorimetric paper sensors for sustainable agriculture

**DOI:** 10.1371/journal.pone.0317739

**Published:** 2025-01-22

**Authors:** Ademir Ferreira da Silva, Ricardo Luis Ohta, Jaione Tirapu Azpiroz, Matheus Esteves Ferreira, Daniel Vitor Marçal, André Botelho, Tulio Coppola, Allysson Flavio Melo de Oliveira, Murilo Bettarello, Lauren Schneider, Rodrigo Vilaça, Noorunisha Abdool, Vanderlei Junior, Wellington Furlaneti, Pedro Augusto Malanga, Mathias Steiner

**Affiliations:** 1 IBM Research, Rio de Janeiro, Brazil; 2 IBM Research, São Paulo, Brazil; 3 Enveritas, New York, NY, United States of America; 4 CSEM Brasil, Belo Horizonte, Brazil; 5 Omnia Fertilizers, Bryanston, South Africa; 6 Integrada, Londrina, Brazil; University of Jeddah, SAUDI ARABIA

## Abstract

For optimizing production yield while limiting negative environmental impact, sustainable agriculture benefits from real-time, on-the-spot chemical analysis of soil at low cost. Colorimetric paper sensors are ideal candidates, however, their automated readout and analysis in the field is needed. Using mobile technology for paper sensor readout could, in principle, enable the application of machine-learning models for transforming colorimetric data into threshold-based classes that represent chemical concentration. Such a classification method could provide a basis for soil management decisions where high-resolution lab analysis is not required or available. In tropical regions, where reliable soil data is difficult to acquire, this approach would be particularly useful. Here, we report a mobile chemical analysis system based on colorimetric paper sensors that operates under tropical field conditions. A standard smartphone equipped with a dedicated software application automatically classifies the paper sensor results into three classes—low, medium, or high soil pH—which provides a basis for soil correction. The classification task is performed by a machine-learning model which was trained on the colorimetric pH indicators deployed on the paper sensor. By mapping topsoil pH on a test site with an area of 9 hectares, the mobile system was benchmarked in the field against standard soil lab analysis. The mobile system has correctly classified soil pH in 97% of test cases, while reducing the analysis turnaround time from days (soil lab) to minutes (mobile). By performing on-the-spot analyses using the mobile system in the field, a 9-fold increase of spatial resolution reveals pH-variations not detectable in the standard compound mapping mode of lab analysis. We discuss how the mobile analysis can support smallholder farmers and enable sustainable agriculture practices by avoiding excessive soil correction. The system can be extended to perform multi-parameter chemical tests of soil nutrients for applications in environmental monitoring at marginal manufacturing cost.

## Introduction

The United Nations Sustainable Development Goals highlight the need for technological innovations to support the sustainable increase of agricultural production [1]. The guidelines are well aligned with precision agriculture practises, which use data-driven management systems based on a set of technologies such as real-time sensing and monitoring for optimizing the use of resources and for improving crop yields [2]. By providing timely and precise characterization of soil parameters, precision agriculture can improve crop productivity while reducing cost and environmental damage [3–5]. Numerous reviews of technology for precision agriculture have been published covering the required components and techniques, from sensors and data processing techniques, to communication networks and actuators [6–8]. Many relate to remote sensing which is less labor intensive and covers large areas [8–19]. However, they rely on extensive soil mapping databases or calibrated models for each soil type and geography, and they are influenced by environmental factors.

For agricultural decision-making at field scale, chemical analysis of soil samples is essential [20, 21]. In precision agriculture, agronomists routinely collect soil samples which are transferred to specialized labs with dedicated equipment operated by trained experts. The process can be time consuming and expensive. In some cases, it might even involve cross-border soil shipments with regulatory complexities. Soil portable analysis kits used for on-site chemical monitoring could provide a much-needed alternative, but often require specialized optical sensors [20, 22], making them impractical or unaffordable for farmers with an annual production equivalent of a few hundred US dollars, as is often the case in agriculture-based economies [23–25]. As a result, smallholder farmers may be cut off from the benefits of up-to-date chemical data needed to improve their agricultural production program.

Paper-based, chemical soil sensors with smartphone-enabled readout could address this need in the field. However, it is unclear to what extent these sensors can reliably produce threshold measurements, such as low/high soil pH, to enable recommendations for soil correction. If implemented, the approach could help avoid excessive use of soil correction product with potentially negative impact on the environment. Also, it could conserve natural resources due to the efficient management of soil properties. Due to the low cost, it could be deployed by smallholder farmers as well as by large agricultural operations, benefiting sustainable agriculture at scale.

Colorimetric paper sensors are made of cellulose paper which contain chemical reagents for soil nutrients. Typically, the reagents react upon deposition of soil extract by color change as function of nutrient concentration. The capillary forces within the cellulose substrate transport a liquid soil extract through microscopic channels to the test output region [26]. Several demonstrations of the use of papers sensors in environmental applications have been reported, particularly in low-income regions [27–29]. Their major advantages are low cost, biocompatibility and that they can be operated without external pumps or electricity. As an example, prior research on testing soil nutrients concentration using paper-based sensors reported a benchmark of colorimetric paper strips for assessing nitrate-N and other elements in soil samples collected at 42 different locations in Kennya and Ghana [30]. At the collection sites, the samples were treated following a nutrient extraction protocol and measured with the paper strips, to be later transported and compared to the results from commercial laboratories in the country. The authors found that, despite variations between testing sites, the strips were sufficiently accurate in assessing the nitrate-N concentration of the sample.

A remaining key issue is automatizing the paper sensor readout in the field. This would improve test reliability overall and could, in principle, enable a non-expert to perform the test. From a technological point of view, recent advances in mobile communication systems have made possible the integration of paper-based sensors within a high-tech/low-tech hybrid approach [31–41]. A smartphone, the high-tech device, can be configured to perform the readout and analysis of a colorimetric paper sensor, the low-tech device, without added hardware features and at virtually no cost to the user.

Recently, such an application was demonstrated in fresh water testing with a system comprising a paper-based phosphate sensor and a smartphone for interpreting and transmitting the results [41]. However, testing soil instead of water would require additional research and development effort for establishing an extraction protocol, with added complexity in paper sensor design, sample preparation, and test readout. Moreover, soil testing in the field would require customized sampling approaches for sample collection as the soil conditions may vary significantly across the test site.

The main knowledge gaps are with regards to the demonstration of colorimetric paper sensors for soil outside controlled lab environments and their automated readout with mobile technologies. Such a demonstration could pose challenges under tropical field conditions, considering the prevalent ambient conditions. An important, open research question is if machine-learning techniques can be applied to reliably transform the colorimetric data produced by the paper sensors into threshold-based classifications, such as low/medium/high, from which reliable soil management recommendations could be derived. A successful demonstration could potentially lower the entry barrier for soil testing technologies in emerging economies.

In the following, we demonstrate a system for mobile soil pH testing in the field by leveraging smartphone assisted paper-sensor readout under ambient light conditions [42] and automated colorimetric indicator analysis with machine-learning models [43]. We have selected pH as the target soil parameter to be analyzed in the field under study. This is because soil pH was identified as an agronomic priority in the present case and, therefore, served as representative soil parameter for demonstrating the viability of the mobile test system at field scale. While the mobile system is capable of detecting pH-values in the range of 3–9, see reference [43], the soil of the test site was found to be generally acidic, with an average pH of about 6. We note that the system can be readily extended to measure other nutrients in soil and water by using a suitable choice of colorimetric indicators.

In addition, we demonstrate how the use of GPS services enables spatial resolution down to the level of meters in the field while cloud computing integration enables field data integration, analysis, and visualization of the test results at scale.

## Materials and methods

### Components of the mobile soil pH analysis system

**Fig 1:**
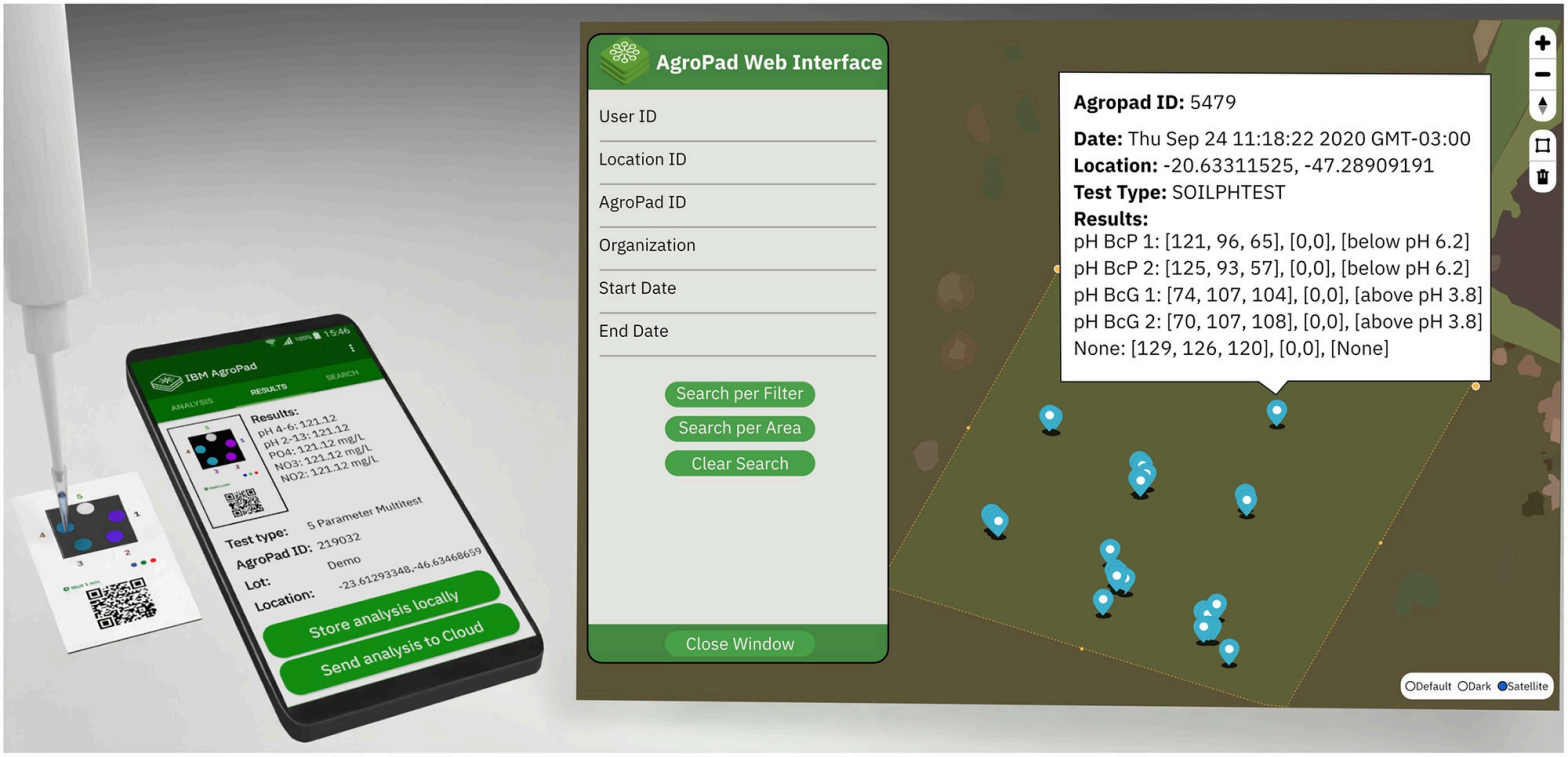
Mobile soil pH analysis system. A liquid sample of soil extract is deposited onto a test card. The test card contains colorimetric indicators for producing color output as function of pH in the soil sample. The sample-specific color output is then analyzed by a mobile application and, by means of integrated AI models, translated into soil pH results. The chemical information is merged with location and time information for local storage or network transfer. (Inset) Web interface for visualizing soil pH test results based on GPS location data. The data is retrieved from a cloud database which integrates the individual measurements performed in the field.

In Fig 1, we show the measurement system developed for this field study. The paper-based sensor with integrated colorimetric indicators reacts upon deposition of a liquid sample of soil extract at the front side and provides a specific color output at the back side that represents the soil pH. A research-grade, non-commercial software application (App) deployed in a representative, commercially available standard smartphone acquires an image of the output layer of the paper device through the phone’s camera. The mobile application, which was developed for the purpose of this study, performs a sequence of analysis steps (image segmentation, color extraction, and execution of colorimetric calibration models) to process the soil pH results. Within seconds, the soil analysis results are available to the user and tagged with time and location information, stored locally on the smartphone, or transferred to a cloud computing platform for data integration, analysis, and visualization. The measured data can be visualized through a web-based user interface.

### Design and manufacturing of paper-based soil pH sensors

**Fig 2:**
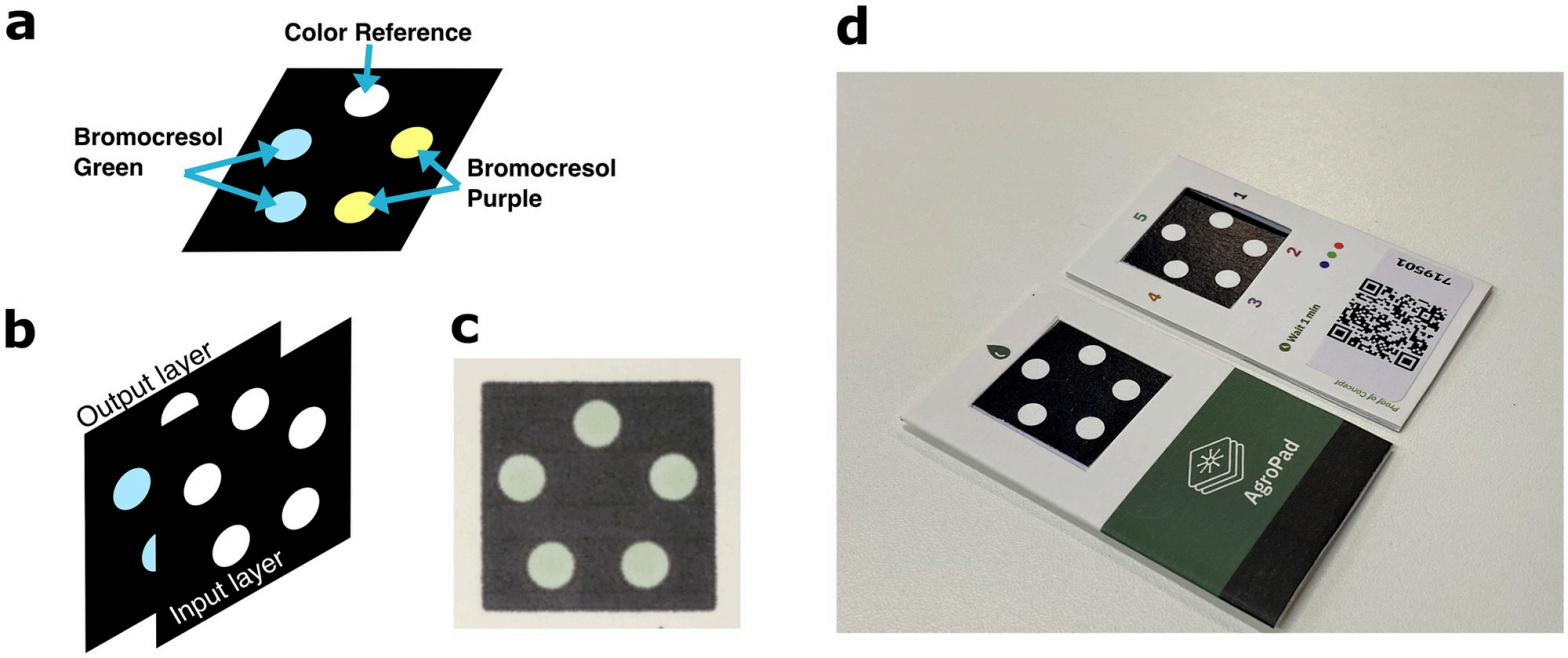
Paper-based, colorimetric soil pH sensor. a) Device layout with colorimetric indicator distribution. b) Assembly of device layers. c) Photograph of the assembled device layers. d) Photograph of the research-prototype sensor, with sample deposition layer (front) and color output layer, reference marks and QR code (backs).

In Fig 2, we show the paper-based soil pH sensor used in this study. Vertically integrated, microfluidic paper-based analytical devices (*μ*-PAD) [26, 44–47] have been successfully demonstrated for water and soil analysis under laboratory condition [27, 35, 48–52]. For field application, we have developed a multi-indicator *μ*-PAD with soil filter function, embedded with color correction references for ambient light correction, and a QR-encoded label for automated processing through the mobile application. The sensor integrates two colorimetric pH indicators, Bromocresol Green (BCG) and Bromocresol Purple (BCP), for creating pH-specific color output.

For manufacturing colorimetric paper sensors for soil pH, we have printed the test layout on two sheets of chromatography paper (3001–861 CHR1 200x200, Whatman) using a wax printer (ColorQube8580, Xerox) as sketched in Figure A of S1 Text. We have then heated the printed layouts at 100°C for 1 minute to allow the wax to impregnate the paper, defining hydrophobic barriers. In a next step, colorimetric indicators are added to the input layer through deposition of 2*μ*L of Bromocresol Green or Bromocresol Purple (Quimlab Inc.) on the wax-defined test spots, as shown in Fig 2a. After a drying step, we have aligned input and output layers and glued them together using a spray glue (Super 77, 3M), as shown in Fig 2b. A stainless steel stencil with a customized design for blocking the test spots has prevented glue deposition on the test regions during process. After cutting each *μ*-PAD square, see Fig 2c, we have packaged it within a cardboard cover containing the test instructions and a color correction reference, as shown in Fig 2d. We have then attached a unique QR code label to each sensor test card containing information with regards to the production lot, the chemical indicators used, and the colorimetric calibration file to be retrieved by the mobile application during test readout. Finally, we have vacuum sealed the test cards in batches of 50 to increase their operational lifespan.

### Mobile phone and cloud computing applications for test data processing

**Fig 3:**
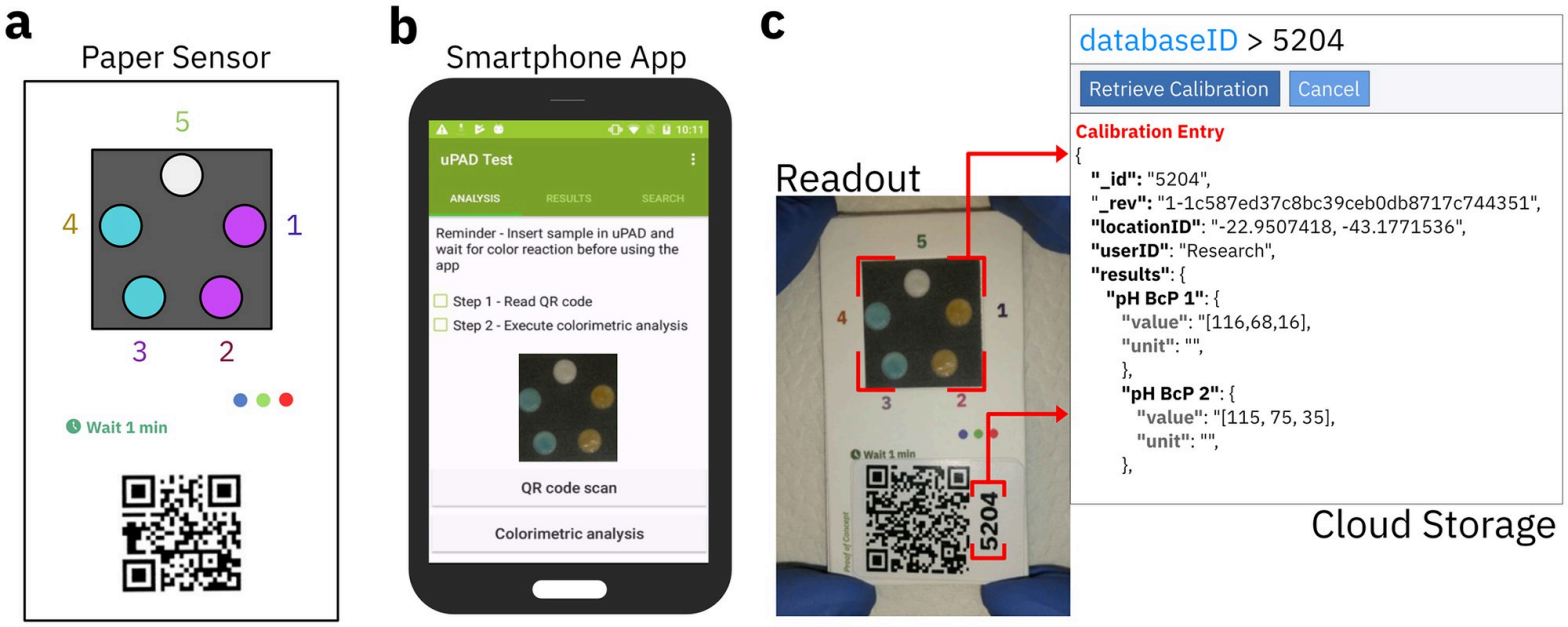
Components of mobile soil pH analysis system. a) Paper-based pH sensor with QR-code and color correction references. b) Screenshot of the smartphone application for test data acquisition, processing, and upload. c) Screenshot of a soil test entry in the cloud computing database.

**Fig 4:**
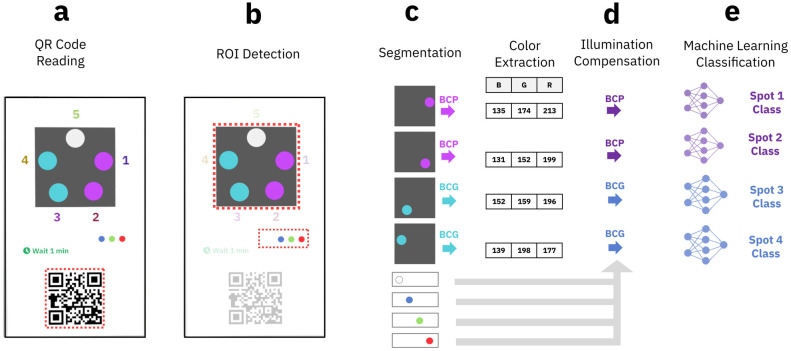
Data acquisition and processing workflow of mobile application. a) Image capture with QR-code readout which is highlighted by dashed red lines. b) Region-of-Interest (ROI) contains sensor output and color correction references as highlighted by dashed red lines. c) Segmentation of colorimetric sensor output spots and color scale references and computation of the mean RGB values for each spot. d) Illumination compensation steps. e) Machine-learning classification step for transforming extracted color data into pH result.

The readout system comprises a mobile application and a cloud application as depicted in Fig 3. In our field study, we have used a representative, standard mobile smartphone (Galaxy S5, Samsung) for test readout. We have processed the acquired images following the workflow depicted in Fig 4 with a dedicated, research-prototype mobile application (operating in Android, 81, Google) developed and implemented specifically for this field study. After readout of a test card, see Fig 3a, the test results together with geolocation data, timestamp, unique ID, and raw images of the test card are immediately available through the user interface of the mobile application, see Fig 3b. The test data can be saved locally, on the smartphone, and, if needed, be transferred via internet connection by means of an API implementation to a cloud based, no-SQL database (Cloudant, IBM). The research prototype cloud application applied in this study was developed specifically to support this project. Fig 3c displays an example of a json-file representing an instance of a soil measurement as saved in the database. Each test entry contains the soil pH result as processed by the mobile application, the colorimetric information of each test point, the geolocation, a timestamp, and a raw image for reference. In addition, we have developed for the purpose of this study a web interface for overlaying all measurements on the map of the test site, enabling improved data access and visualization.

### Test data acquisition and processing workflow

During test readout, the mobile application acquires a single image of the test card and reads the respective QR-code label as shown in Fig 4a. The QR code contains the information with regards to the machine learning model to be used for test processing. After image acquisition, a processing routine, which is visualized in Fig 4b, segments both the paper-based sensor area where the test spots are located, as well as the area containing the reference colors for correction. After segmentation, the RGB values of each test spot are read out, see Fig 4c, and corrected for ambient light and sensor conditions, see Fig 4d, using the color correction references and following the algorithm outlined in reference [42]. Finally, the corrected RGB values are passed on to pre-trained machine learning models for classification into output classes, see Fig 4e.

### Training of machine-learning classification models

We have trained machine-learning classifiers for transforming the RGB color within the sensor images into pH classes by means of the mobile application. Specifically, the calibration dataset contains images of paper sensors tested with liquid soil extract obtained with 14 reference soil samples with pH-values ranging from 3.4 to 6.9, obtained under laboratory conditions prior to field testing.

We have prepared each soil extract by sieving the soil sample through a 2mm mesh sieve, mixing the result with 0.01M CaCl_2_ solution in a 1:2.5 ratio in a vial with a cap, shaking it for 60 seconds and letting it settle for 20 minutes. We have shaken the solution again for a few seconds, waiting 5 more minutes for the formation of the supernatant, as described in section *Development of soil pH extraction protocol* of S1 Text. For reference, we have measured the pH of each soil extract using a commercially available, potentiometric pH meter (Simpla 140, AKSO).

**Fig 5:**
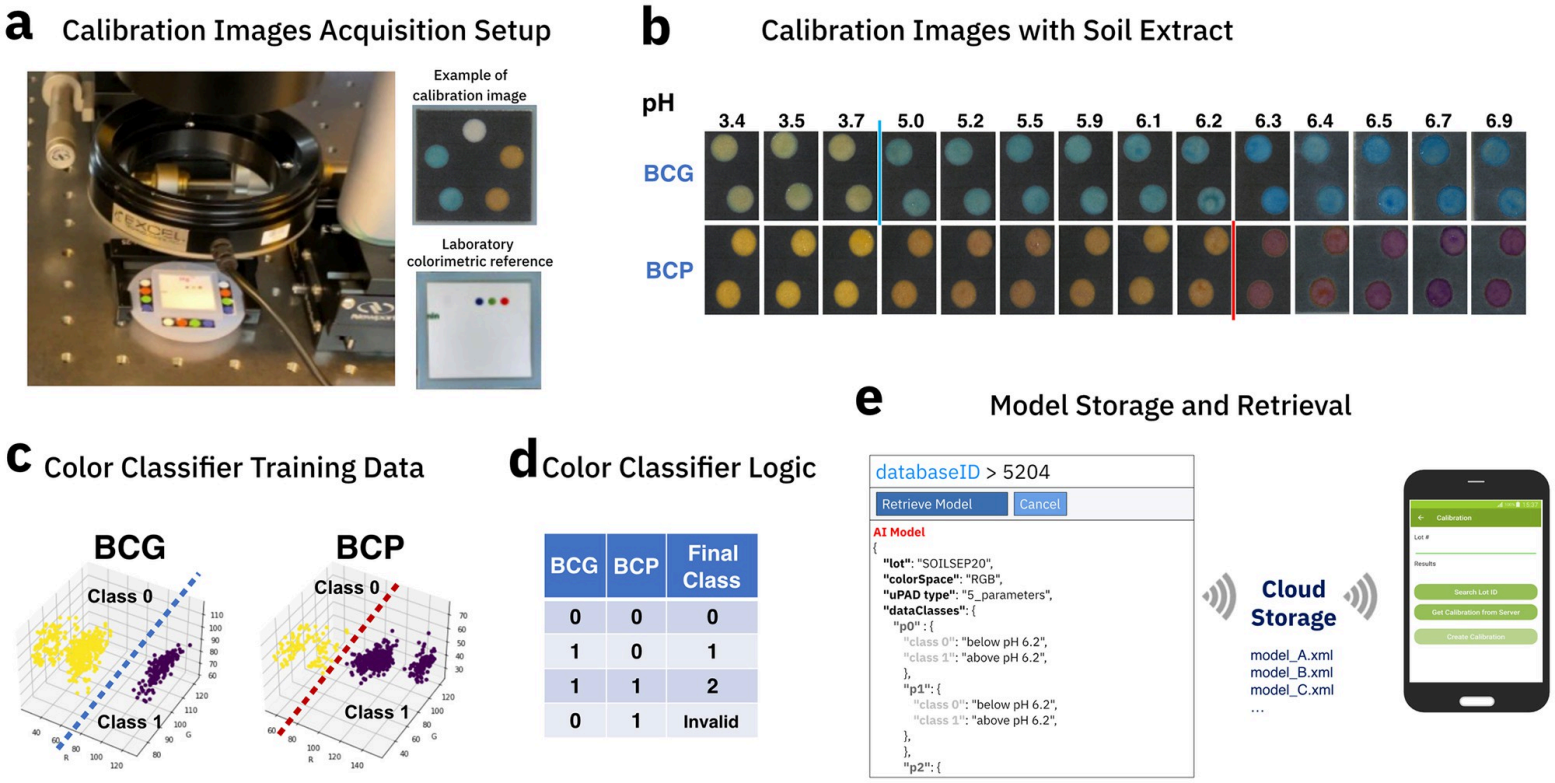
Soil pH calibration data acquisition and processing. a) Experimental lab setup for acquiring colorimetric calibration data. b) Calibration images of soil extracts at various pH levels. c) RGB distribution of measured calibration data. d) Logic for combining the classification results of BCG and BCP colorimetric indicators into pH classes. e) Screenshot of cloud database with calibration model storage and retrieval.

The test protocol starts with pipetting 15 *μ*L of soil extract supernatant onto each test spot and waiting for 5 min. The calibration acquisition setup in Fig 5a consists of a machine-vision camera (PL-D734CU-T, Pixelink) equipped with a telecentric lens (Newport) and a homogeneous illumination source (LED144A, AmScope). Fig 5b shows a set of representative images of the calibration dataset. A colorimetric reference image is also captured using the same setup. It serves as reference for the illumination and hardware correction algorithm of the mobile application [42], see Fig 4d.

Using the same image processing routine outlined in Fig 4c, the tests spots of the calibration images are automatically segmented, the mean RGB value of each test spot is computed and saved as a calibration dataset correlating it to the reference pH value of each sample. We have defined three soil pH classes based on the operating range of the colorimetric indicators with standard pH buffer solutions, see Figure C in S1 Text. A “low pH” class for values below 3.9, a “high pH” class for values above 6.3, and a “medium pH” class for the values in between. The result of both colorimetric indicators measured simultaneously yield the classification of the soil pH into the classes described above. With regards to the pH classification labels, we note that in precision agriculture a pH-value of 7 can be considered “high pH”, see [53]. Following agronomic classification standards we have, therefore, labeled the class with pH-values above 6.3 as “high pH”. Fig 5b and 5c highlight the colorimetric response of both indicators to soil extracts, and Fig 5d shows the color classifier logic used for class definition.

For field testing, we have prepared a calibration dataset with a total of 300 identical paper-based soil pH sensors containing the two indicator spots of BcG and BcP, respectively, as shown in Fig 5a. They were tested with 14 reference soil samples for a total of 600 data points for each indicator. During data processing, each sensor card image undergoes an image processing routine for segmentation and extraction of RGB values representing each test spot. In a subsequent processing set, instances that exhibited segmentation issues or outlier RGB values, i.e., 1.5 times above or below the interquartile range, are removed. Finally, the processed calibration dataset contains 590 and 560 data points for BcG and BcP, respectively. As shown in Fig 5c, the BcG data is separated into 160 and 430 data points for class 0 and 1, respectively. In the case of the BcP indicator, the distribution is 460 data points for class 0 and 100 for class 1.

The RGB and HSV values of all data points have served as feature vectors for model training. We have applied a 5-way cross-validation training routine, splitting the calibration dataset randomly by 80:20, and averaging the accuracy score. We have used the OpenCV library (https://opencv.org/) as it is suitable considering the computational limitations of mobile devices, supports offline operation at locations that lack network connectivity, and enables the exchange of model parameters between platforms via XML files, as illustrated in Fig 5e.

During field testing, we have used a logistic regression model with an accuracy of up to 85% relative to the calibration dataset in the mobile application, after having imported the model parameters from an XML file. In addition to the XML file containing the model parameters, for each test card lot, we have merged the information about the chemical indicators, the classification labels, the test card lot ID, and the color correction references into json-files. As depicted in Fig 5e, the files are stored in a cloud database from which the mobile application can retrieve the respective model during operation. The workflow allows for model updates without changing the mobile application.

The machine-learning classification models, as well as the python scripts used in data preparation and model calibration, are available at [54]. Also, the data used for model calibration is available at [55].

### Soil sample collection and test preparation in the field

**Fig 6:**
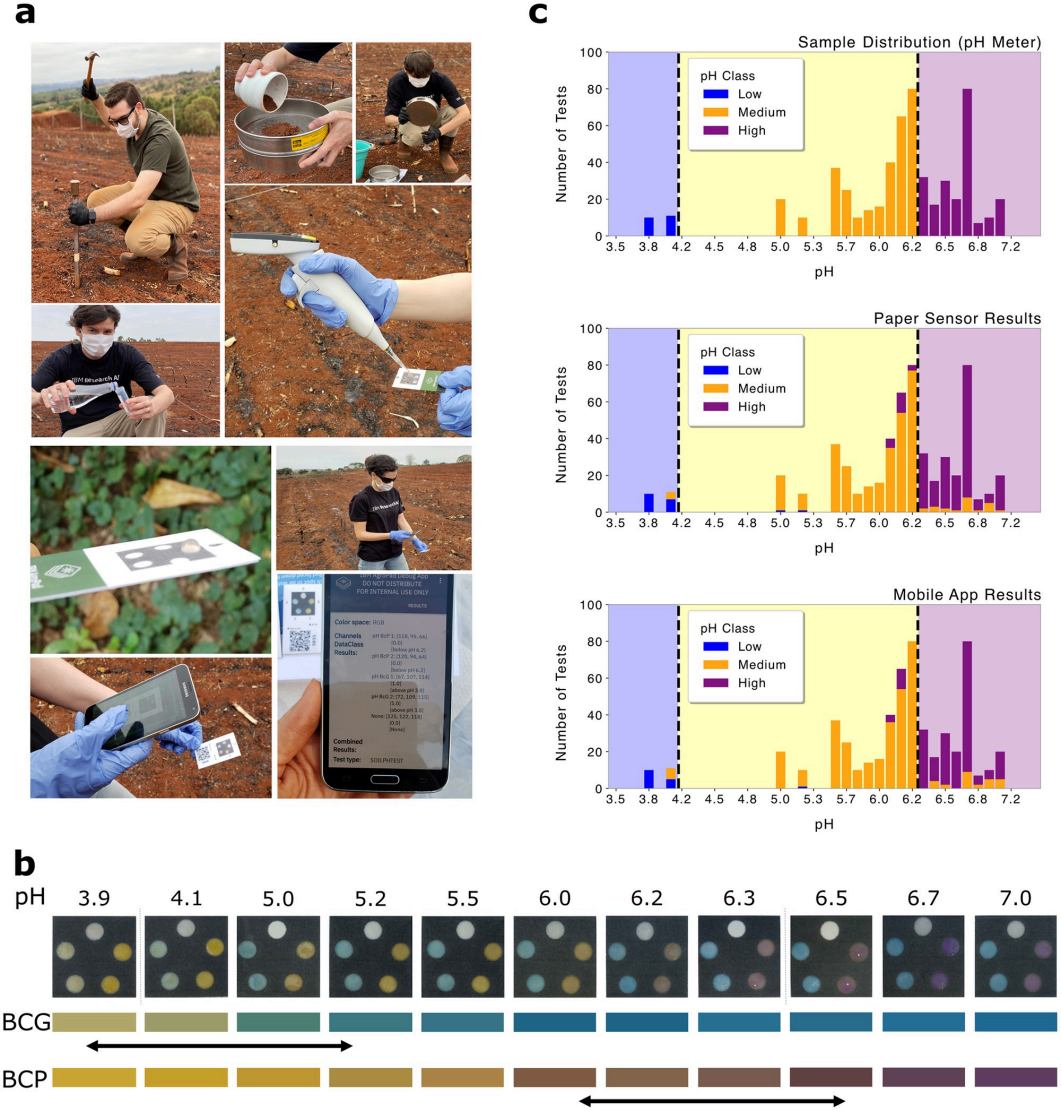
Mobile soil pH analysis in the field. (a) Soil sample collection, processing, and smartphone assisted readout of the test card in the field. (b) Representative photos of the output layer area of the paper sensor depicted in (a) as collected in the field. Depending on the pH value of the deposited soil sample, the output layer features the characteristic color of integrated colorimetric indicators (circles 1, 2: BCP; circles 3, 4: BCG, circle 5: reference w/o indicator). The bar charts visualize the colorimetric outputs obtained in the different pH regimes. (c) Soil pH field data. Upper panel: Number of soil pH occurrences for all samples included in this study (N = 548). Medium panel: pH classification results as per visual analysis of the test card output. Lower panel: pH classification results as per automated, mobile application assisted analysis of the test card. The dashed lines separate classification results with regards to low pH (pH 3–3.9), medium pH (pH 4.0–6.3), and high pH (pH 6.4–9).

At each demarcation location, we have inserted a soil probe about 20 cm deep into the ground for extracting a soil sample, see Fig 6a. Overall, we have collected a total of 81 soil samples from the sampling zones over the course of three days.

During the field test, we have applied for each soil sample the same soil extraction method used during the calibration procedure, i.e.; 1) sieving the sample through a 2mm mesh sieve, ii) mixing the result with 0.01M CaCl_2_ solution in a 1:2.5 ratio in a vial with a cap, iii) shaking it for 60 seconds and letting it settle for at 20 minutes, iv) shaking the solution again for a few seconds and waiting 5 minutes for the formation of the supernatant. For more details, we refer to section *Development of soil pH extraction protocol* in S1 Text.

### Mobile soil pH analysis in the field

For evaluating the reproducibility and accuracy of the mobile soil pH analysis system, we have tested soil samples by using three independent methods: the colorimetric paper-based sensor, a standard pH meter, and by means of independent soil laboratory analysis.

For the colorimetric tests, we have deposited 15 *μ*L of the soil extract supernatant on each test spot of the paper sensor. We have set the sample volume and reaction time to 15 *μ*L and 2 minutes, respectively, to guarantee that the colorimetric reaction has completed and to minimize reflection-induced variability in the image data caused by excess liquid. After 5 minutes of wait time, we have acquired an image of the test card output using the mobile application.

Within seconds, the App displays the pH classification of the soil sample and stores the test result on the smartphone (see Fig 6a). The data is streamed to the Cloud database as soon as an internet connection is available. Fig 6a illustrates the field application workflow of the mobile measurement system for soil analysis. Each soil extract sample is measured 10 times with different test cards. After the mobile soil pH analysis, each soil extract sample is further tested using a potentiometric pH meter (Simpla 140, AKSO). Representative photos of the output layer area of the paper sensor as captured in the field are displayed in Fig 6b spanning the range of pH values observed in the samples. The bar charts visualize the mean colorimetric outputs obtained in the different pH regimes for each integrated colorimetric indicators (circles 1, 2: BCP; circles 3, 4: BCG, circle 5: reference w/o indicator).

The field test data are available at [55].

### Statistical data analysis

We have analyzed the pH classification results obtained using standard performance metrics, namely the distribution of correct and incorrect predictions per class and per dataset, respectively, as well as estimates of precision, recall, and F1-score, each per class and per dataset. The accuracy of the model is defined as the number of correct predictions (true positives and true negatives) divided by the total number of predictions. As an indicator of the quality of the prediction, we have determined the precision of a classification as the number of true positives divided by the total number of positive results (true or false). We have determined recall performance as the number of true positives divided by the sum of true positive and true negative results. This measure provides the sensitivity of the classifier by assessing the proportion of positive class samples present in the dataset that are correctly identified by the model. Finally, we have computed the F1-score as the harmonic mean of precision and recall, i.e. 2*(precision*recall)/(*precision* + *recall*). The F1-score has a maximum value of 1.0, indicating perfect precision and recall, and is especially useful in the analysis of unbalanced class datasets, where classes have different numbers of samples.

## Results and discussion

**Fig 7:**
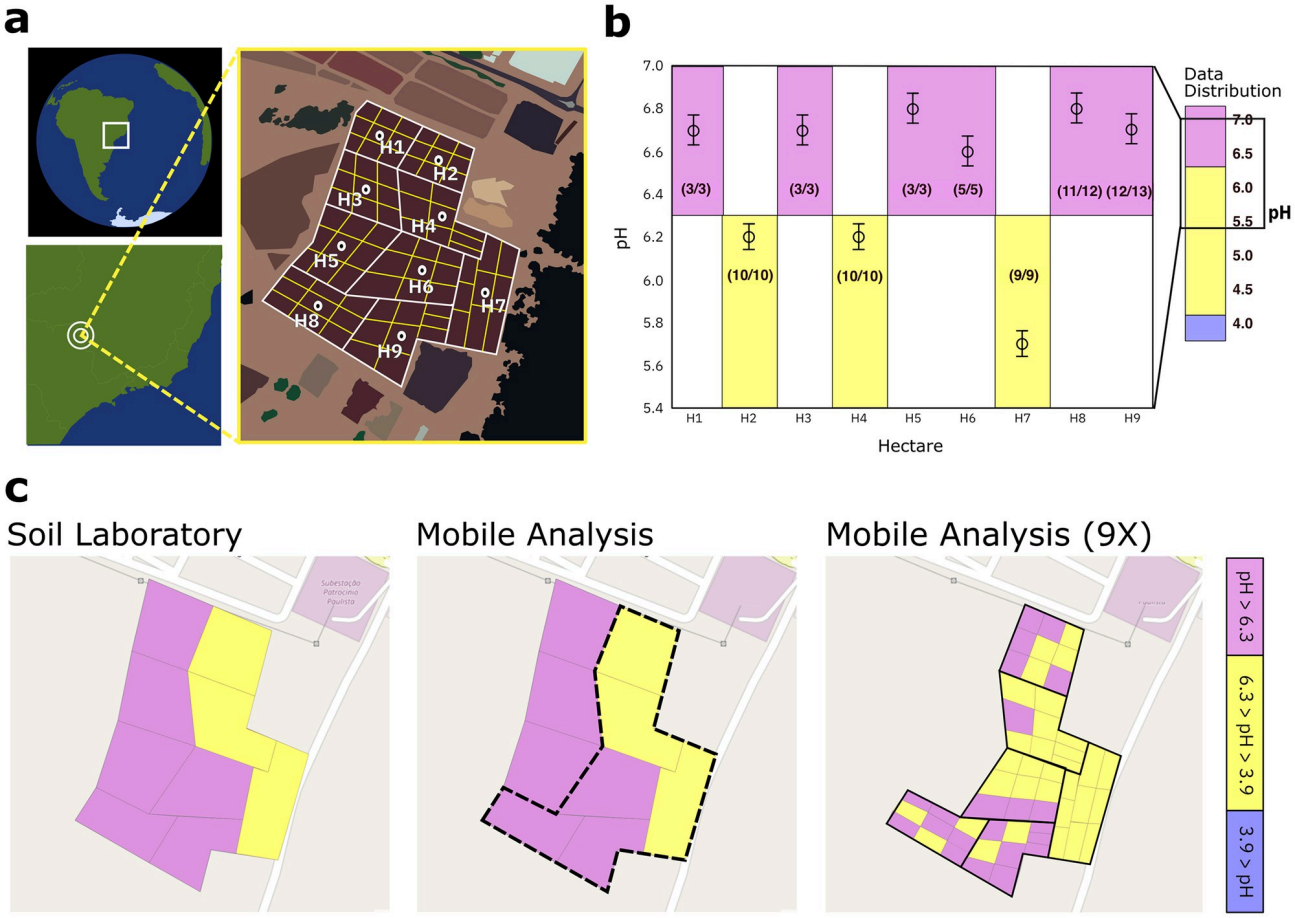
Spatial distribution of soil pH in the field. (a) Geographical sketch of the testing site located at -20.6342520 latitude and -47.2905210 longitude, and demarcation of soil sample collection zones labeled H1-H9. (b) Soil pH results obtained with the mobile system in the field and reference results obtained by a soil lab from the same compound samples collected in H1-H9. The graphic on the right-hand side highlights the relevant pH-range, 5.4–7, and the pH threshold at 6.3 which separates the classes “medium pH” and “high pH”. The symbols represent the pH of the compound samples as measured by the soil lab, and the number in parenthesis represent the ratio between correct mobile field test results and total number of mobile field tests performed for a specific compound sample. (c) Spatial map of soil pH visualizing the compound sample pH classification derived from soil lab analysis data (left) and pH classification of the same samples obtained with the mobile soil pH analysis system (middle). The bold dashed lines (middle) delineate the area for which soil samples were analyzed with 9x higher spatial resolution as compared to standard protocol. The paper sensor results (right) reveal the spatial fine structure of soil pH distribution at the test site.

The test site is a 9-hectare area of a soybean farm at an altitude of 750 meter above sea level located in Patrocinio Paulista in the state of São Paulo, Brazil. Prior to sample collection, we have delineated the test area into 9 cells, each with an area of about one hectare each, as indicted by the white lines in Fig 7a. We have then further subdivided each cell into 9 sampling zones, geo-tagged the zones, and marked them with yellow flags.

Overall, we have performed a grand total of 805 colorimetric paper-based sensor tests to confirm the accessible measurement range and accuracy of the mobile soil pH analysis and successfully detected instances within all three pH classes. This includes 615 tests performed in the field and 190 tests performed under laboratory conditions. To ensure the accuracy of the results, we measured the pH of all 81 collected soil samples with a pH meter as well.

Both colorimetric indicators are simultaneously interrogated for soil pH below (Class = 0) or above (Class = 1) the respective colorimetric turning point. By combining the classifications of the two indicators, we have defined the pH class shown in Fig 5d. Accuracy is determined by whether the correct pH class was predicted with the paper-based test.

For separating the error analysis of test card and mobile phone application, we have performed a visual inspection of each test card by a human expert to verify if the color output represents the actual pH value of the sample. Based on the visual inspection, we obtain an overall colorimetric paper-based sensor accuracy of 73% (590 Out of 805). This means that, on average, about three out of four test cards have developed a proper colorimetric reaction and produced a valid test result. The overall smartphone assisted readout accuracy of the test cards is 72% (579 Out of 805).

Upon closer examination, we have observed that 180 out of 615 measurements from 20 out of the 54 sampling zones (soil samples) could have potentially been impacted by the effects of premature sample evaporation, suggesting that testing parameters such as sample volume and reaction time need to be properly adjusted to the weather conditions (see Section *Evaluation of field test measurements and soil pH extraction protocol* in S1 Text). A subset of 77 additional data points that yielded the correct classification result were still discharged based on the date of collection as those tests were also deemed compromised. For analyzing potential accuracy improvements with refined measurement conditions, we have replaced the compromised field measurements by a set of measurements repeated on the same soil samples in our lab. The corrected data set contains 548 test results. The plots in Fig 6c show the distribution of the 548 paper-based test results with regards to soil pH as established by the reference measurement with the pH-meter. The upper panel displays the number of soil pH occurrences for all samples included in this study. The medium panel and lower panels display the pH classification results as per visual analysis of the test card output and as per the automated, mobile application assisted analysis of the test card, respectively. The dashed lines separate classification results with regards to low pH (pH 3–3.9), medium pH (pH 4.0–6.3), and high pH (pH 6.4–9).

If we perform the smartphone assisted readout with the subset of test cards that have produced a proper colorimetric output as confirmed by visual inspection, we obtain an accuracy of 92% (505 Out of 548). On average, about nine out of ten properly functioning test cards are read out and classified correctly by the mobile application, attesting to the App’s reliability of both colorimetric calibration and ambient light correction. We conclude, therefore, that the mobile soil pH analysis overall accuracy is currently limited by the test card performance.

**Fig 8:**
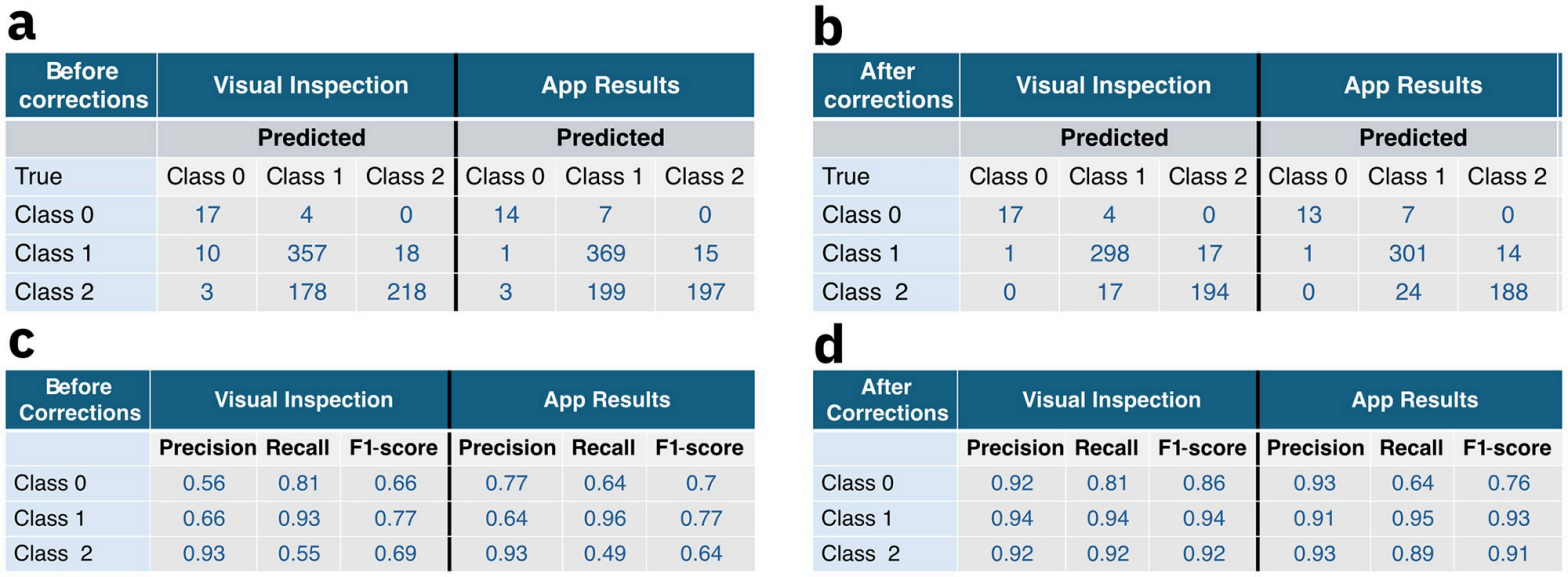
Distribution of pH classification results and metrics. Distribution of Predicted class of each test result with regards to the True class representing the label of the soil sample applied to the paper sensor of the a) initial data set with 805 test points and b) the processed data set with 548 test points. precision, recall, and F1-score per class of the c) 805-point dataset and d) 548-point dataset.

To assess the performance of the classification system, we have analyzed the classification statistics and compared the results with the reference soil pH data. Fig 8 displays the distribution of predicted pH classes obtained by performing the test, denoted as *Predicted*, separated with regards to the *True* class representing the label of the soil sample applied to the paper sensor. For comparison, Fig 8a displays the distribution of test results following both visual interpretation and mobile App readout in the initial dataset with 805 tests while Fig 8b displays the distribution of the processed dataset containing 548 tests. In addition, Supplementary Figure Ha and Hb in S1 Text display the confusion matrices corresponding to the data in Fig 8a, while Supplementary Figure Hc and Hd in S1 Text show the confusion matrices corresponding to the data in Fig 8b.

We observe that most misclassifications in Fig 8a and Supplementary Figure Ha and Hb in S1 Text have occurred for predicted class 1 if testing a sample that corresponds to true class 2. The proportion of misclassifications within true class 2 is of the order of the correctly predicted tests, with slightly larger values in case of mobile App readout as compared to the visual inspection case. The corresponding true and predicted class results for the post-processed data in Fig 8b and Figures H c and d indicate that the laboratory repetitions were able to address misclassifications of class 2 as class 1 on both visual and App results, with the proportion of incorrect predictions significantly reduced. Test results for true class 0 and 1 remain largely unchanged in both datasets, with a proportion of correct predictions per class ranging from 64% to 95%. Supplementary Figures E and G in S1 Text show the distribution of total test accuracy across the pH range of the initial dataset and the processed dataset, respectively.

A similar trend can be observed in the classification metrics precision, recall, and F1-score for each class as presented in Fig 8c and 8d for both the initial and the processed dataset, respectively. The average metrics yields an overall 71% precision, 76% recall and 71% F1-score with regards to the visual inspection results, and a 78% precision, 70% recall and 71% F1-score with regards to the App readout results of the 805-point dataset. Similarly, the corresponding metrics for the corrected, 548 point dataset has produced and averaged 92% precision, 89% recall and 91% F1-score for visual inspection, and an averaged 92% precision, 83% recall and 87% F1-score for the App case, respectively. It follows that the data correction significantly improved the misclassification of true class 2 as class 1 on both visual and App readout. We, therefore, conclude that improving the robustness of the mobile system against external weather conditions can ensure accuracy and precision values above 90%.

In a next step, we have compared the paper-based soil analysis with precision agriculture standard reference. To that end, we have produced one compound soil sample for each hectare by combining the nine soil samples taken from each of the sampling sub-zones. The compound samples were then shipped to a soil analysis laboratory (Ribersolo) for independent analysis.

With the paper-based mobile soil analysis, we have determined the pH class of each cell or sub-cell by the majority of all paper test measurement results on the same soil sample, that is, a class 1 result means that 50% or more of the paper-based measurements had produced a class 1 outcome.

As a key result of our study, we find that our mobile soil pH analysis result correctly predicts the pH class obtained with the soil lab results for all nine cells investigated, resulting in 6 cells with “high pH”, 3 cells with “medium pH”, and zero cells with “low pH” as displayed in Fig 7c left and middle plots. As the pH of the soil varied between 5.5 and 7.0, only two of the three pH classes occur on the maps, as expected. Through repeat measurements, we find that the mobile system differentiates correctly between “medium pH” and “high pH” in 66 out of 68 cases, leading to an overall measurement accuracy of 97%.

Note that Fig 7c right displays the spatial distribution of the paper-based test results in the data set collected from the field, after they were corrected for compromising weather conditions with repeat lab measurements of the same samples. For comparison, we show in Figure Ia in S1 Text the spatial distribution of pH-values as measured by the pH-meter using the classification scheme shown in Fig 5d.

We would like to point out that we have independently validated the pH values obtained with the field-based soil extraction method. As shown in Supplementary Fig D in S1 Text, we have measured the pH of all soil extracts using a reference method with a standard pH-meter and we have observed quantitative with the pH results obtained from the soil analysis lab.

We now investigate if a spatial resolution enhancement of the pH mapping can potentially reduce the use of soil correction product, thus enabling sustainable agriculture practices. For 6 of the 9 cells studied, we have performed mobile soil pH analysis at each of the GPS locations directly in the field. The results provide a mapping of soil pH at higher spatial resolution as compared to the compound sample analysis performed at cell level in Fig 7c left and middle. We find that the chemical mapping at higher spatial resolution reveals pH variations not visible in the compound analysis. The enhanced resolution can potentially improve soil health management efficiency: If pH corrections were to be performed based on the 9x resolution mapping, an additional 16 out of the total 54 sub-zones, or 30% of the area, would be treated accurately as compared with the mapping in 1-hectare standard resolution. By assuming soil treatment would be performed to increase pH from “medium pH” to “high pH”, about 22% of soil correction product could be saved for a total of 6 subzones that would not require soil pH correction in this scenario. The resolution advantage could particularly benefit farmers with smaller fields and limited resources for soil correction.

The main limitations of the current methodology are identifying robust, low-cost colorimetric indicators for important soil parameters, as well as the application-specific training and adoption of machine-learning classifiers to each soil parameter and agricultural program, respectively. This requires, to some extent, progress in model training automation along with the development of a centralized model data base. Another limitation is with regards to the mobile phone hardware, because transferability of the application will require progress in image sensor calibration and light correction algorithms. Finally, the paper sensor layout and materials should be further improved for limiting sample extract evaporation and maintaining measurement conditions in the field.

Looking forward, we note that the colorimetric paper-based sensor system can potentially integrate a broad range of colorimetric indicators for adaption to specific use cases [32, 34, 35, 39, 41, 46, 48, 49, 52]. As a multi-parameter sensor example adapted to agricultural requirements in Southeast Brazil, we show in Figure J in S1 Text a paper sensor prototype that, in addition to pH, integrates colorimetric indicators for the detection of Magnesium, Calcium, and Aluminum ions. Future research work requires the development of colorimetric calibrations and soil extraction methods to support field-scale benchmarking of a broader class of soil nutrients and chemical parameters. Considering the rising adoption of smartphones globally, mobile soil pH analysis could soon become a technologically viable, low-cost alternative to lab testing in emerging economies.

## Conclusion

We have reported the first AI-enhanced mobile soil pH analysis with colorimetric paper sensors at field scale. The method produces reliable soil pH classification results in near real-time for low-cost applications in sustainable agriculture. The time for performing a mobile soil pH test in the field is of the order of 30 mins, mainly due to sample collection and soil extract preparation. This is much shorter than the turnaround time for standard lab analysis ranging from days to weeks, depending on infrastructure. As described in section *Manufacturing of colorimetric paper-based sensor* of S1 Text, we have performed manufacturing scaling studies for analyzing the efficiency of the colorimetric paper-based sensor production workflow. We estimate paper sensor manufacturing costs of about US$3.00 per piece at the current R&D scale (thousands of devices) and below US$0.25 per piece at larger production scale (millions of devices).

Based on our results, a smartphone user could now conceivably perform a soil test with a colorimentric paper-based sensor at a pre-defined GPS location, retrieve the chemical results immediately to perform remediation action locally and, finally, stream the measured test data to a database for spatio-temporal analysis. This would improve agricultural efficiency while, at the same time, excessive use of chemical product and the related environmental damage could be avoided. Besides the time advantage in obtaining chemical results in the field, soil sample shipments could be limited to cases where high-resolution data and in-depth agronomical analysis are required. The application of machine-learning models to the analysis of the colorimetric sensor results enables capturing features in the indicator response to variations in the soil extract composition that visual inspection may not be able to capture, potentially improving the overall test quality. With sufficient training data, the calibration process could be further simplified and automated, thus relying less on human expert intervention.

## Field study permission

Engagement of entities and individuals participating in the study was covered by evaluation agreement #B1873809/Request ID 37099 which were reviewed through the governance and compliance processes of the IBM Research Contracts organization. Written informed consent for site access, soil sample collection, and soil chemical testing was provided by the owner of the field test site. Photo consent forms for the researchers appearing in the manuscript figures were obtained prior to manuscript submission. All individuals appearing in the manuscript figures are also co-authors of the manuscript.

## Supporting information

S1 Text Supplementary information. (PDF)
