# Supplementary material for: AI enabled, mobile soil pH classification with colorimetric paper sensors for sustainable agriculture
Source: PLoS One. 2025 Jan 22;20(1):e0317739. doi: 10.1371/journal.pone.0317739 (PMC11753690; doi:10.1371/journal.pone.0317739)
Supplement: S1 Text — (PDF) [file pone.0317739.s001.pdf]

AI enabled, mobile soil pH classification with colorimetric paper sensors for sustainable agriculture

-SUPPLEMENTARY INFORMATION-

Ademir Ferreira da Silva[1], Ricardo Luis Ohta[2], Jaione Tirapu Azpiroz[1], Matheus Esteves Ferreira[1], Daniel Vitor Marçal[1], André Botelho[2], Tulio Coppola[2], Allysson Flavio Melo de Oliveira[2], Murilo Bettarello[3], Lauren Schneider[3], Rodrigo Vilaça[4], Noorunisha Abdool[5], Vanderlei Junior[6], Wellington Furlaneti[6], Pedro Augusto Malanga[6], Mathias Steiner[1*]

[1] IBM Research, Rio de Janeiro, Brazil, [2] IBM Research, São Paulo, Brazil; [3] Enveritas, New York, USA; [4] CSEM Brasil, Belo Horizonte, Brazil; [5] Omnia Fertilizers, Bryanston, South Africa [6] Integrada, Londrina, Brazil

* mathiast@br.ibm.com

Manufacturing of colorimetric paper-based sensor

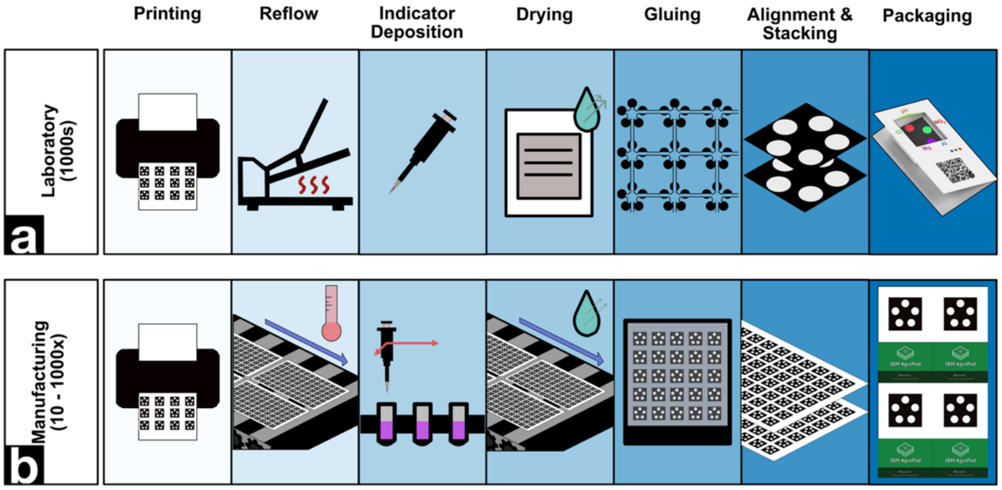

Figure A. Manufacturing steps for the production of paper-based, colorimetric sensors. Production steps at **a)** laboratory scale and at **b)** industrial scale.

To explore the scaling potential of colorimetric paper-based sensor manufacturing, we have performed a production study which is visualized in Figure A. While the wax printing step remains

essentially unchanged, the main manufacturing bottlenecks occur in the deposition of the chemical indicators and in the device assembly. In our study, we have considered the same class of wax printers used in lab-scale manufacturing to print the test layout on paper. We note that parallel operation of multiple printers is possible.

During the reflow phase, the wax-printed sheets are heated as they travel through an in-line reflow oven. For deposition of liquid indicators, we insert the paper sheets into automated reagent deposition equipment. The sheet drying step is carried out in another in-line oven set to a lower operating temperature. We deposit glue by means of serigraph printing, where liquid glue is pressed through a stainless-steel mask to avoid exposure of the reaction zones. The sheet alignment and stacking steps are carried out with the help of fiducial markers or holes in the paper sheets. Once aligned and sandwiched, the sheets are cold pressed to ensure the contact between the layers is homogeneous. Finally, individual μPADs are cut out at sheet level with a cut and crease machine.

We have evaluated all the above steps on the production floor, except for automated pipetting of indicators. The cardboard covers are manufactured following standard scaled printing and cutting process while QR code stickers are added at sheet scale. Individual μPADs are then aligned between sheets of front and back cardboard covers, glued, and pressed in place before vacuum sealing the devices either individually or in bundles for distribution.

The production program we have explored for a 2-layer paper-based device with an area of $24x24mm^2$ is detailed in Table A. Assuming an 8-hour production period within an industrial manufacturing environment, the program would enable a monthly production of +300.000 devices, considering one production period per day, one set of machines, 22 working days and using the bottleneck process as a reference.

**Item**	**Category**	**Process Description**	**Machine Description**	**Estimated Production Rate (Per min)**	**Estimated Sensor Production**	
					**Per hour**	**Per 8hrs**
1	Paper Sensor	Printing	Wax Printer	10 sheets	10800	86400
2		Channel formation	Reflow Oven	1 meter	5400	43200
3		Reagent deposition	Automated Liquid Handling	300 depositions	2160	17280
4		Sheet drying	Reflow Oven	1 meter	5400	43200
5		Gluing	Screen Printer	5 sheets	10800	86400
6		Alignment & Stacking	Customized Equipment	3 sets	6480	51840
7		Lamination	Roll laminator	10 sheets	21600	172800
8		Cutting	Cut & Crease Machine	6 sheets	12960	103680
9	Cover	Printing	Digital Press	120 sheets	115200	921600
10		Cutting	Cut & Crease Machine	6 sheets	11520	92160
11		Gluing	Glue Machine	20 meters	89600	716800
12	Assembly	Chip alignment & cover stacking	Customized Equipment	1 set	1920	15360
13		QR Code	Label Printer	6 meters	43200	345600
14		Packaging	Vacuum Sealer	4 bags	2400	19200

Table A. **Program for scaled manufacturing of paper-based, colorimetric sensors**

Development of soil pH extraction protocol

The analysis of soil through colorimetric reactions requires extracting soil nutrients from soil samples in liquid phase by means of chemical reagents. In the application of colorimetric paper-based sensors, the papers' cellulose fibers need to withstand the chemistry used for nutrient extraction.

Various extraction methods exist, each with parameters that can influence the colorimetric response of the reaction. Some examples are:

I. Soil chemical property to be analyzed (pH, calcium, magnesium, organic matter, etc.).
II. Type, volume, and concentration of the extractor solution.
III. Volume of the soil sample.
IV. Extraction time.
V. Tools to be used.
VI. In the case of soil acidity, the soil buffering capacity [1-3].

Buffering capacity refers to the ability of soil to resist changes in pH and increases with cation exchange capacity and organic matter content [2]. This parameter requires particular attention for ensuring consistency between the true value of soil acidity and the colorimetric result. In practice, the pH stabilization of the liquid sample of soil extract can take several minutes, depending on soil pH and buffering capacity. Significant pH variations typically occur within 15 minutes after the soil sample is mixed with the extractor.

For our field study, we have followed the extraction protocol for measurement of soil pH issued by EMBRAPA [3]. The protocol consists of sieving the sample through a 2mm mesh sieve, mixing it with 0.01M $CaCl_2$ solution in a 1:2.5 ratio in a vial with a cap, shaking it for 60 seconds and letting it settle for at least 15 minutes. To test and validate the extraction protocol for application within the paper device, we have used a set of reference soil samples from various locations in Brazil. The samples were collected from topsoil, down to 20 cm below the surface, dried in natural air and sieved following the same protocol for consistency. After mixing the soil samples with a $CaCl_2$ solution of pH=5.5, pH measurements were taken with a pH-meter (Simpla 140, AKSO) at distinct time intervals. In Figure Ba, we plot the average pH value obtained from 10 soil pH extraction procedures performed with the same soil sample, jointly with the respective standard deviations. Based on the results, we have chosen an extraction time of 20 minutes to optimize the trade-off between extraction time and pH readout robustness.

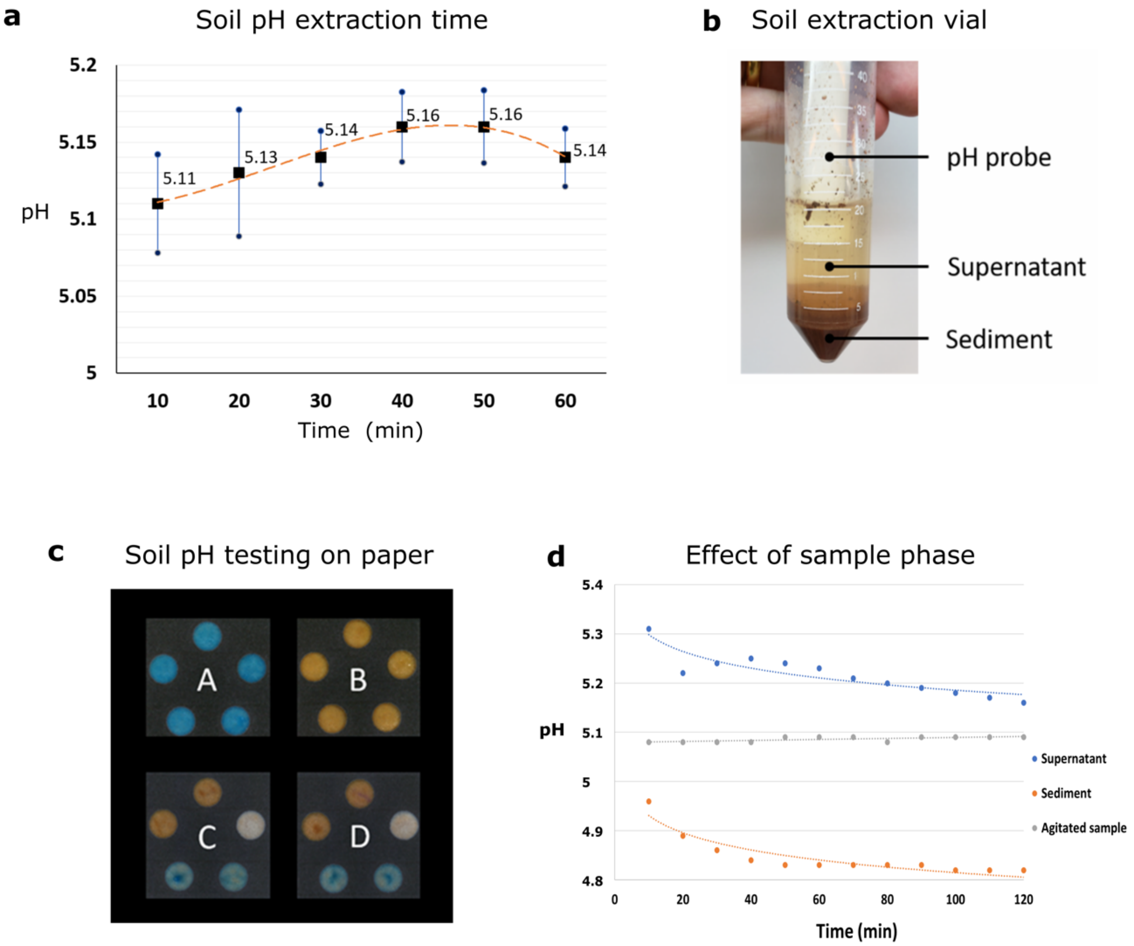

Figure B. Soil sample extraction protocol. a) Measured pH of soil extract as function of time. The dashed line is a guide to the eye. **b)** Soil extract formation with a region of particulate matter at the bottom of the solution and a supernatant region on top. **c)** Influence of the presence of particulate matter on colorimetric test results. **d)** Measured pH as function of time in the supernatant, sedimental, and agitated sample phase, respectively. The dotted lines are guides to the eye.

The use of the soil extraction protocol in combination with the colorimetric paper-based sensors requires further consideration. During extraction in the vial, the soil solution forms regions of particulate matter at the bottom and supernatant at the top, see Figure Bb. The particulate matter could potentially cause clogging of the flow pathways and influence the color formation in the paper sensor device. This effect is shown in Figure Bc: 40μL of extract were deposited on each test spot and have reacted with the colorimetric indicators for a few minutes. Specifically, extract from the supernatant region was deposited on the sensors shown in Figure Bc, A and B, having the same reagent Bromocresol Green (BCG) and Bromocresol Purple (BCP), respectively, on all 5 test spots. In both cases, we obtain a homogeneous color distribution. For comparions, extract from the sedimental region was deposited on two devices impregnated with BCP and BCG on two spots each, see Figure Bc, C and D. Due to the higher concentration of particulate matter in the sedimental sample, the colorimetric output of those devices exhibit stains in the test output areas.

To mitigate the above issues, we have added to the soil extraction protocol an additional step in which the sample is shaken after 20 minutes of extraction time, followed by an additional 5-minute wait time for the formation of the supernatant. A small amount of soil extract is then pipetted from the supernatant region onto the test spots. We observe that this procedure reduces the pH difference in the soil extract between the various phases of the soil extract and mitigates the negative influence of the particulate matter on the test result, see Figure Bd.

Based on the above, the field test soil extraction protocol was refined as follows:

1. Collect a soil sample with a soil sampler or probe.
2. Sieve the soil sample through a 2mm-mesh sieve.
3. Mix 2.5mL of 0.01M $CaCl_2$ solution (pH=5.5) for each mL of sieved soil in a plastic vial and close the vial with a cap.
4. Shake the solution for 60 seconds and wait for 20 minutes.
5. Shake the solution again for a few seconds and wait 5 mins for the formation of the supernatant.
6. Collect the supernatant with a pipette and deposit 15 µl on each test spot of the paper sensor.

Following the above protocol, for each soil sample, we have typically produced about 25 mL of extract solution.

Colorimetric calibration with pH buffer standard references

We have determined the operating range of the colorimetric indicators by testing the paper-based sensors with a total of 31 standard pH buffer solutions, ranging from pH 3.0 to pH 9.0, with a step size of 0.2 pH (Quimlab Inc.).

We have measured each solution 10 times with paper sensors from the same lot, using the acquisition setup shown in Figure 5a of the main manuscript. For each datapoint, we have processed the absolute RGB values measured with the experimental setup by means of principal component analysis and the most significant component of each reagent is shown in Figure C as a function of pH.

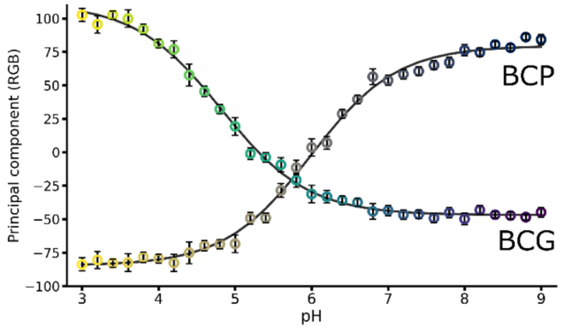

Figure C. Colorimetric calibration curve obtained with pH buffer standard references. Principal components of measured color output (symbols: experimental data, lines: sigmoid fit functions) obtained for colorimetric indicators Bromocresol Green (BCG) and Bromocresol Purple (BCP), respectively, using pH buffer standard references across the full pH range of the colorimetric paper-based sensor.

Evaluation of field test measurements and soil pH extraction protocol

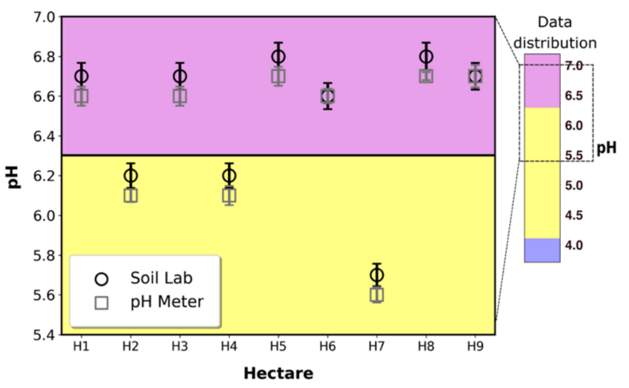

Figure D. Benchmark against soil laboratory analysis. Soil compound sample pH-values obtained with the pH-meter in our lab (using the same extraction method applied in the field) and results for the same compound samples retrieved from the external soil laboratory.

For establishing a reference data set, we have measured all collected soil samples with a commercially available pH-meter (Simpla 140, AKSO), directly on the test site. We have then created one compound samples or each hectare by combining nine samples taken from each of the sampling sub-zones, see Figure 4b of the main manuscript. One portion of each compound sample was sent to an external soil analysis lab and another portion was sent to our own laboratory for reference measurement with the pH-meter. Figure D plots the pH values retrieved from the soil lab and those obtained from the same compound samples by using the pH-meter in our lab, showing agreement. The results confirm that the soil extraction protocol we have applied in our study yields pH results consistent with standard soil lab analysis.

In Figure E, we show the distribution of 805 colorimetric paper-based sensor results with regards to soil pH as established by the reference measurement with the pH-meter. The accuracy is determined by whether the correct pH class was predicted with the paper sensor test card. For each colorimetric paper-based sensor, two data points were extracted by processing the outcome of the four test spots, in pairs of one BCG and one BCP each, following the logic in Table B.

pH class	Bromocresol Green	Bromocresol Purple
Low pH (< 3.9)	0	0
Medium pH (3.9 < pH < 6.3)	1	0
High pH (> 6.3)	1	1

Table B: pH class definition with regards to the colorimetric result on the paper sensor

The results were then benchmarked against the pH-meter measurements. We have binned the pH data in increments of 0.2 and within each bin, the green fraction represents the proportion (0 to

100%) of correct classification while the red represents the proportion of incorrect ones. Figure Ea displays the test measurement accuracy according to a visual interpretation of the colorimetric result on the test card performed by an expert user and Figure Eb displays the results of the classification model retrieved from the mobile application. Overall, the binned results in Figure E demonstrate that the mobile soil acidity analysis correctly predicted classifications consistent with the measurements by the pH-meter in over 70% of the cases. The results show a reduced classification accuracy for samples with a pH-value close to the class boundary occurring at pH=6.3, in both visual inspection of the colorimetric response as well as automatized mobile application classification. Improving the classification accuracy at the class boundary could be achieved with further refinement and optimization of the measurement protocol and with the addition of complementary colorimetric indicators on the paper sensor.

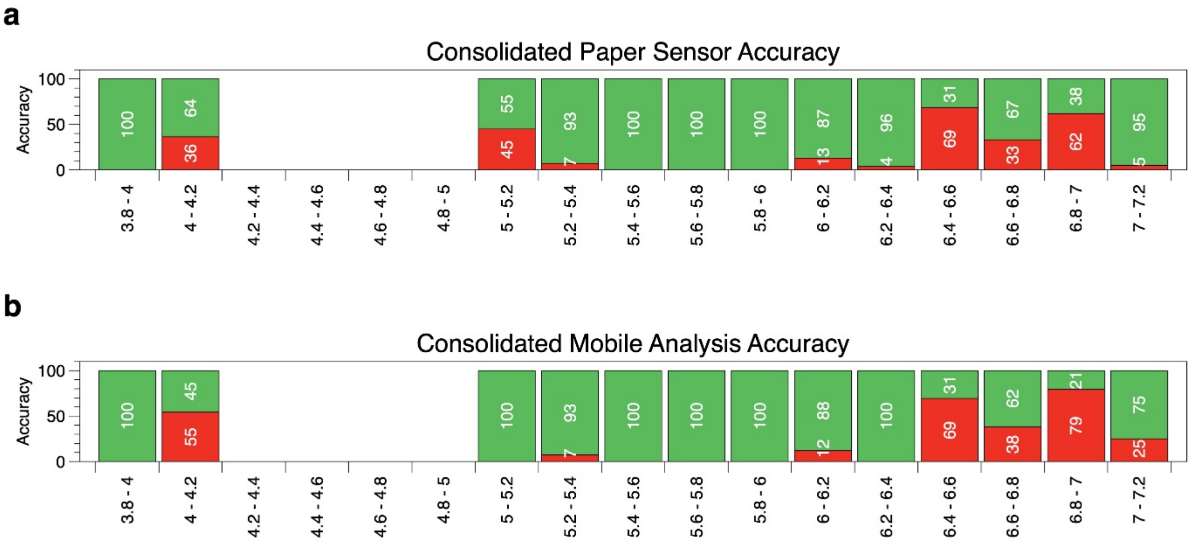

Figure E. Soil pH distribution of paper-based sensor as confirmed with the pH-meter. Green fractions represent the correct classifications, red fractions represents incorrect classifications. The test numbers of each fraction are indicated in white. **a)** Accuracy of visual interpretation of the colorimetric output on the paper sensor by an expert user. **b)** Accuracy with model applied through mobile application.

By analyzing the colorimetric reaction dynamics of the BCP indicators within a paper sensor under laboratory conditions, we have observed that at the class boundary of pH= 6.3 the sensor requires significantly longer reaction times for producing a robust color output. Figure Fa displays the reaction dynamics. At a pH-value of 6.2, the time for achieving a robust color output raises to above 125 seconds, whereas it remains well below 50 seconds away from this value. The results indicate that the test accuracy at the class boundary could be improved by optimizing the wait time before the readout is performed.

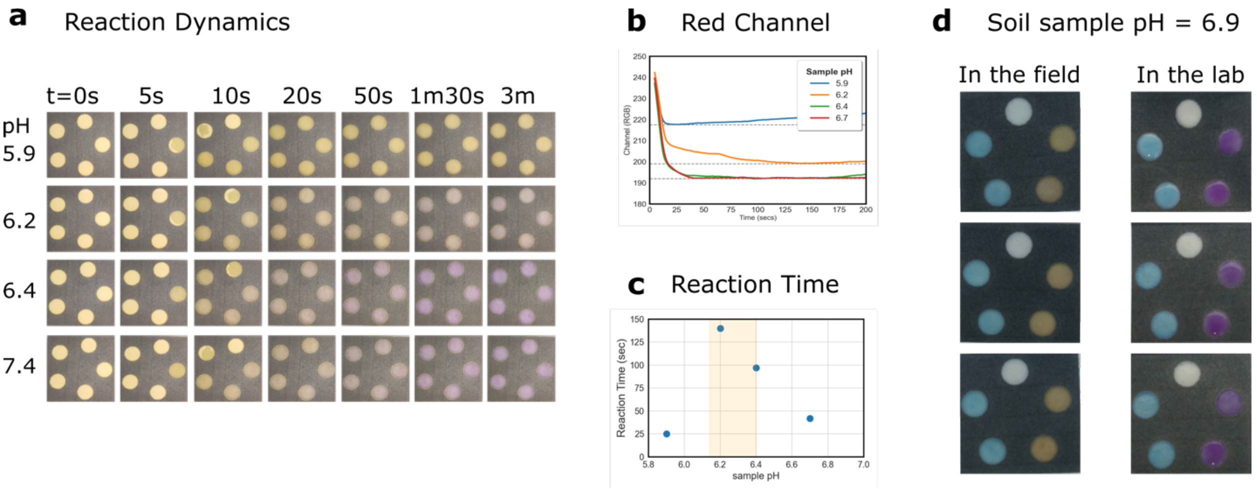

Figure F. Analysis of colorimetric reaction dynamics. **a)** Colorimetric reaction dynamics measured on four paper-based sensors. All sensor test spots treated with the BCP indicator exhibit temporal variations. **b)** Time evolution of camera recording in the red channel averaged across five sensor spots on each paper device. **c)** Colorimetric reaction times extracted from the evolution of the dynamics observed in the red channel in **b**. **d)** Color formation with the same soil sample extract at pH=6.9 performed (left) in the field - suffering premature sample evaporation - and (right) in the lab.

In addition, we have investigated potential misclassifications in the pH range of 6.3-7.0 in field measurements that were impacted by the varying weather conditions experienced during the field test. Those measurements were expected to produce a colorimetric response; however, they did not develop well, mainly due to high temperature, around 35°C, as compared with the lab temperature of 22°C. The high temperature in the field has led to premature evaporation of the liquid sample of soil extract, prior to completion of the colorimetric reaction. This effect is shown in Figure Fd. We conclude that both the volume of sample as well as the wait time should be adjusted to account for weather conditions. We have found that 180 out of 615 measurements from 20 out of the 54 sampling sites could have potentially been compromised by the effects of premature sample evaporation. In addition, we have discharged a subset of 77 additional data points that yield the correct classification result based on the collection date as those tests might have been compromised as well. We note that adjusting the sample volume to 15µL and the reaction time to 120s should ensure proper test operation even at elevated temperatures above 30°C.

For analyzing the potential accuracy improvement with refined measurements conditions, we have replaced the compromised field measurements by a set of measurements repeated on the same soil samples in our lab and the accuracy results are plotted in Figure G. As compared with the results shown in Figure E, the corrected data set containing 548 test results clearly displays an improved classification accuracy at the class boundary of pH=6.3, as well as an improvement in the range between 6.3 and 7.0, due to avoidance of premature sample evaporation.

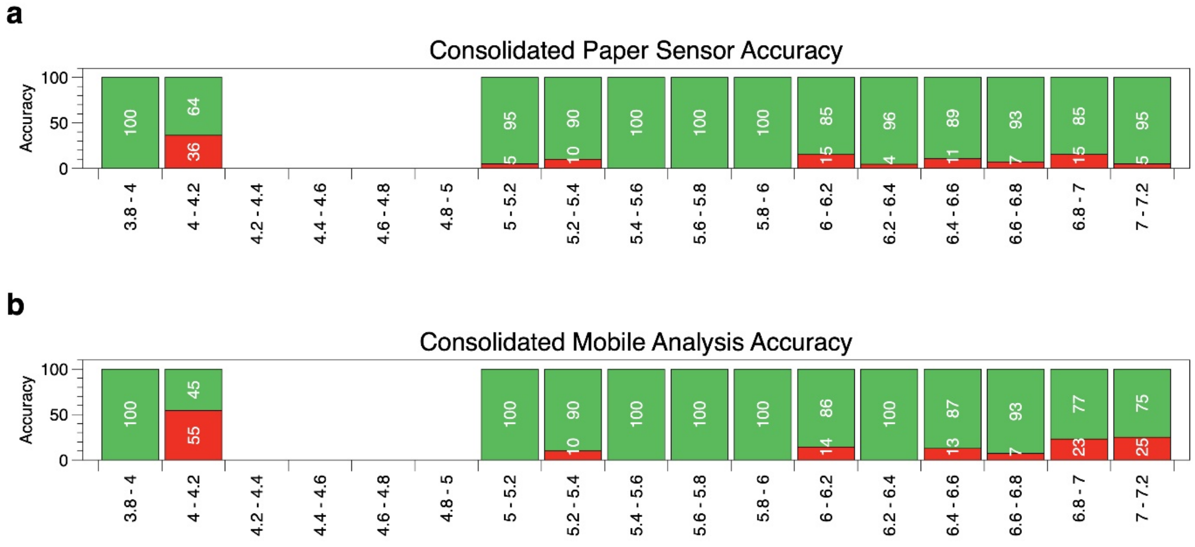

Figure G. Soil pH distribution of paper-based sensor as confirmed with the pH-meter. Green fractions represent the correct classifications, the red fractions represent incorrect classifications. The test numbers of each fraction are indicated in white. As compared to the data set shown in Figure E, about 180 field measurements compromised by weather conditions were replaced by laboratory repetitions. **a)** Accuracy of visual interpretation of the colorimetric output on the paper sensor by an expert user; **b)** Accuracy with model applied through mobile phone measurement.

We have further performed a statistical analysis of the system performance and the smartphone-assisted readout of the paper sensor output displayed in Fig. 6, 7 and G. We have collected the distribution of classification results in Figure 8 of the main manuscript according to the predicted class obtained after performing the test, denoted as *Predicted*, and separated based on the *True* class representing the measurement of the corresponding soil sample applied to the paper sensor. Figure 8a displays the distribution of test results following both visual interpretation and mobile App readout on the original dataset prior to processing with 805 tests. Figure 8b displays the distribution on the processed dataset with 548 tests. Figures H a and b display the confusion matrices corresponding to the data in Figure 8a, as computed by the Python library Scikit-learn [4] and normalized to the total number of tests per *True* class (matrix row) in the original 805 points dataset for the visual inspection and App readout results, respectively. Figures H c and d show the corresponding normalized confusion matrices resulting from the data shown in Figure 8b for the processed 548 points data set. These confusion matrices provide an assessment of the accuracy of the classification where the values along the diagonal represent the proportion of correct classifications per true class and the off-diagonal values indicate to the proportion of incorrect classifications within each class.

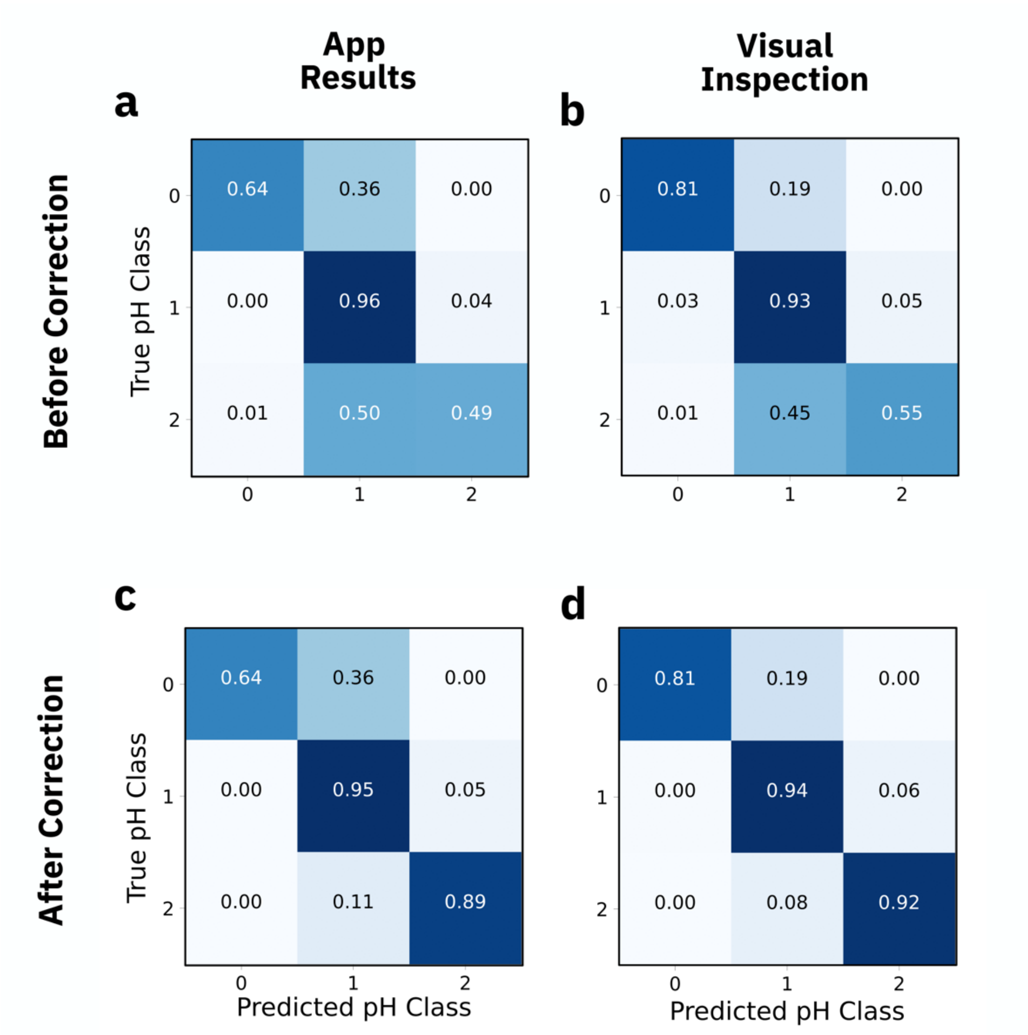

Figure H: Confusion matrix representation of the soil pH classification results. Confusion matrices normalized to the total number of tests per *True* class (per row) of the pre-processed 805-point dataset after a) mobile App classification and b) visual inspection, and of the processed 548-point dataset after c) mobile App classification and d) visual inspection.

Analysis of compound soil samples

To analyze the accuracy of the paper-based, colorimetric sensor results in the field, see Figure 7 of the main manuscript, we have grouped the pH-meter results in three classes according to the logic laid out Table B, that is, "low pH" for soil pH below 3.9, "medium pH" for soil pH between 3.9 and 6.3, and "high pH" for soil pH above 6.3. The spatial distribution of soil pH-values obtained by measuring with the pH-meter and using the above classification scheme is displayed Table B. The pH of the soil on the test site varied between 5.5 and 7.0. Therefore, only results within two of the three pH classes occur on the map. When compared with the results of compound sample results in Figure 7c of the main manuscript, due to the higher spatial resolution, we observe a higher spatial variability of pH-values. Figure Ib displays the pH class distribution as measured

by the paper-based sensor cards for the dataset collected in the field. For each sample, we have determined the pH class through majority of ten test card measurement results on the same soil sample, that is, a "high pH" result means that 50% or more of the test card measurements have produced a "high pH" classification outcome. Overall, we have obtained 13 misclassifications in 51 zones, corresponding to a classification accuracy of 75%. Figure Ic shows the paper-based test results after they were corrected for compromising weather conditions (premature sample evaporation) with repeat lab measurements performed on the same soil samples. As a result, in Figure Id we have obtained an improved classification accuracy with only three misclassifications in a total of 54 zones, boosting pH classification accuracy to 94%.

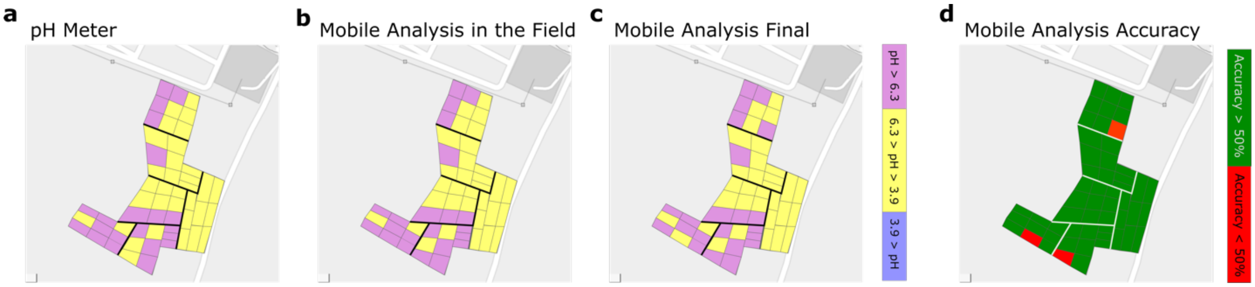

Figure I. Spatial mapping of soil pH classification results in the field. a) Spatial distribution of the soil pH as measured by the pH-meter and grouped in three classes, that is, "low pH" for soil pH below 3.9, "medium pH" for soil pH between 3.9 and 6.3, and "high pH" for soil pH above 6.3. **b)** pH class distribution as measured by the paper-based, colorimetric sensor in the field. The result is determined per majority of 10 test outcomes per cell. **c)** Corrected pH class distribution after replacing measurements compromised by weather conditions with lab measurements on the same soil samples. **d)** Paper-based sensor accuracy map, after replacing measurements compromised by weather conditions with lab measurements on the same soil samples.

Multi-Parameter Test Prototype

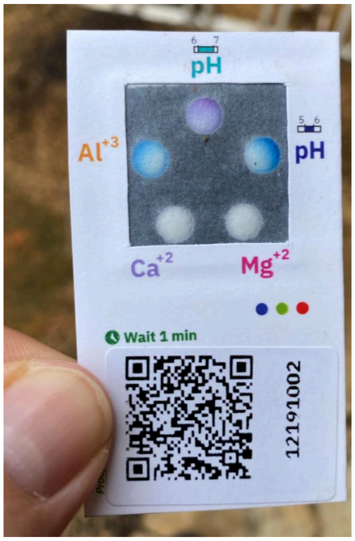

Figure J. Paper-based sensor prototype integrating colorimetric indicators for the simultaneous detection of soil pH, Aluminum, Calcium and Magnesium ions.

References

[1] M. Motsara and R. Roy, *Guide to laboratory establishment for plant nutrient analysis*, (Vol. 19). Rome: Food and Agriculture Organization of the United Nations, 2008. https://www.fao.org/3/i0131e/i0131e.pdf

[2] P. Hazelton and B. Murphy, *Interpreting soil test results: What do all the numbers mean?*, Melbourne: CSIRO Publishing, 2007. https://doi.org/10.1111/sum.12402

[3] P. C. Teixeira, G. K. Donagemma, A. Fontana and W. G. Teixeira, *Manual de métodos de análise de solo*, Brasília, DF: Embrapa, 2017. https://ainfo.cnptia.embrapa.br/digital/bitstream/item/181717/1/Manual-de-Metodos-de-Analise-de-Solo-2017.pdf

[4] Pedregosa *et al.*, Scikit-learn: Machine Learning in Python, JMLR 12, pp. 2825-2830, 2011.
